# Supplementary material for: Measuring the popularity of football players with Google Trends
Source: PLoS One. 2023 Aug 16;18(8):e0289213. doi: 10.1371/journal.pone.0289213 (PMC10431623; doi:10.1371/journal.pone.0289213)
Supplement: Attachment — Submitted filename: Measuring the popularity of football players with Google Trends.docx [file pone.0289213.s004.docx]

Dear author(s):

Thank you for the opportunity to read your manuscript "Measuring the popularity of football players with Google Trends". The study uses a dataset comprised of 1428 football players from five European leagues during the 2018-2019 season and calculates popularity indicators to measure the popularity of these football players and the contribution of popularity to predict players’ market value.

Please find my detailed comments below to aid you in the further development of your study.

**Abstract**

- The abstract should be more straight forward and could need a bit of restructuring to make it clearer and to have a better understanding at first glimpse. What is the research question, what are the methods used, what are the results and findings, as well as the implications?

**Introduction**

- You write that “The sports industry is a scenario where most interests converge, from political to public, increased by communication media and information.” I am not sure I understand this sentence. Could you reformulate it please?
- What exactly do the authors refer to when mentioning “income”? Club revenues, Gross income, net income? Player income?
- You state “Football club revenues are often closely related to teams' investment in their main assets: the players”, as has been previously pinpointed by Frick (2007). But what do you exactly mean with this sentence, that clubs with higher revenues are able to purchase players that have higher transfer fees?
- Since you study a probable effect of “popularity” on “market value”, you should first of all properly assess the two key variables, i.e. popularity and market value. Popularity is quite detailed described in section 1.2. As of “market value” you cite Herm et al. (2014) with “an estimate of the amount of money that a club would be willing to pay for an athlete contract, regardless of an actual transaction”. However, this definition has been criticized in the past. What “club” do Herm et al. refer to? Does the term “market” not rather suggest that we are not considering one club but several clubs or several stakeholders? How do we know what a single club is willing to pay if they do not communicate it? And in negotiation process or even after, it is highly unlikely that a club would communicate the prize they would have been willing to pay. Given that in your study you do not ask one or several clubs for their willingness, but rather obtain your market values from transfermarkt, you should be consistent here. A discussion of transfer and market value has been provided in a recent working paper by Franceschi et el. (What can football economics literature learn from subjective value theory?). They also provide an overview of alternative definitions in their appendix.

**Background**

- The section is rather short and the summary of previous research performed in the three described parts (Player characteristics, Player performance, and Player popularity) is not elaborately enough and too unspecific. I suggest you explain the findings in much more detail and the partly conflicting results of different existing studies. The results are not as unitary as you make it sound. Please have a look at Franceschi et el. (2023) for a recent literature review on football player valuations.

*Ref*

*Franceschi, M., Brocard, J. F., Follert, F., & Gouguet, J. J. (2023). Determinants of football players’ valuation: A systematic review. Journal of Economic Surveys.*

- You write that “Researchers have identified three principal groups of variables for analysing a player's market value: Player characteristics, Player performance, and Player popularity.” Since you site Frick (2007) you are aware that there are a few more factors. You could further add a section for other influencing factors that cannot be summarized under these three categories but that have been found to influence market value in the past, such as current market conditions, current and future clubs, leagues of the selling and purchasing club, the nationality of a player – in fact, some of these factors could be factors that influence market value via your variable popularity. A player that has been playing for a relatively short time at a club outside Europe and is suddenly purchased by a top club receives a big boost in terms of popularity (latest example Endrick from Palmeiras who will be transferred to Real Madrid in 2024).
- In the section on Player characteristics, you refer to previous analyses that have found contract length to influence performance. What do you want to tell the reader here? Is remaining contract length a player characteristic? And why do you write about performance as an endogenous variable here?
- You write that “while the characteristics and performance of players have helped predict their transfer fees, they are not perfect measures of a football player's market value, so complementing them with popularity data could offer a better idea of a player's valuation.” This assertion falls somewhat from the sky and you do not refer to previous research nor have a comprehensible rationale for this claim. Please elaborate.
- You write that “…the brand image is also sold or exchanged with the player…it is necessary to study the specific effect of popularity on the transfer fee.”

The concept of image rights, as understood today, stems from the so-called “right of publicity” (Al-Ameen, 2017). However, the ownership and control of image rights, which can be exploited through sponsorship, endorsement and merchandising, is not subject to legal rules on allocation of rights and responsibilities but is a mere question of negotiations between clubs and players (Haynes, 2007). It is common practice that clubs enter into employment contracts with their athletes that require them to cede the use of their brand or image for marketing and commercial activities (Hewison, 2001). While a few powerful football club such as Arsenal, Manchester United and Real Madrid demand full control over players’ image rights, other clubs are just content to get a minimal share of the proceeds from the commercial exploitation of the player’s image rights (Al-Ameen, 2017). Star players in top leagues strive to circumvent the image right obligations by, often successfully, leveraging their position to obtain advantageous conditions, whereas average players are often denied this privilege.

*Ref.*

*Al-Ameen, H. A. (2017). Image right clauses in football contracts: masterstroke for mutual success? Intellectual Property Rights, 5(1).*

*Hewison, J. (2001, 07.12.2001). Legal teams take to the football field to thrash out standard player's contract. Retrieved 10.08.2022 from* [*https://www.lawgazette.co.uk/news/legal-teams-take-to-the-football-field-to-thrash-out-standard-players-contract/35706.article*](https://www.lawgazette.co.uk/news/legal-teams-take-to-the-football-field-to-thrash-out-standard-players-contract/35706.article)

- Some rather recent articles using machine learning technics in determining market value have not been considered at all, such as
- *McHale, I. G., & Holmes, B. (2022). Estimating transfer fees of professional footballers using advanced performance metrics and machine learning. European Journal of Operational Research.*
- *Yigit, A. T., Samak, B., & Kaya, T. (2020). An XGBoost-lasso ensemble modeling approach to football player value assessment. Journal of Intelligent & Fuzzy Systems, 39(5), 6303-6314.*
- *Zhang, D., & Kang, C. (2021, April). Players’ Value Prediction Based on Machine Learning Method. In Journal of Physics: Conference Series (Vol. 1865, No. 4, p. 042016). IOP Publishing.*

**Materials and methods**

- What was the reason you only included players of the big 5 leagues? Maybe you could perform the analysis by additionally using players of other popular leagues such as the Eredivisie, German Bundesliga 2, Portuguese Primeira Liga, and Belgium First Division where there is enough information available and the players are known, thus do not cause problems with market value. Additionally, these are the leagues where a lot of players of the Big 5 leagues are bought from. This way you would have a bigger dataset than that of 1428 players, which is a rather small dataset (as you pointed out yourself in your concluding remarks) – sample size is important, especially in a machine learning context and the degrees of freedoms you have.
- Do you have examples of other studies where the model has been trained with one data set (in your case market values) and then tested with another dataset (transfer fees)?
- As for the dependent and explanatory variables used, there might be the need to add a variable indicating player injuries. Injuries are very common in the game of football, and while short to midterm injuries have little impact on market values, they certainly impact virtually all player performance variables.
- Previous research has found evidence for customer and employer-based discrimination based on nationality in the case of football (Pedace, 2008). Players from highly populated and football enthusiastic countries such as Mexico and Brazil might have distorted popularity figures when comparing these figures to their market values. One such case was James Rodriguez when playing for Bayern Munich between 2017 and 2019. In your study you do not control for nationality or home population

*Ref*

*Pedace, R. (2008). Earnings, performance, and nationality discrimination in a highly competitive labor market as an analysis of the English professional soccer league. Journal of Sports Economics, 9(2), 115-140.*

- You used 5 different leagues and the market values as provided by transfermarkt with two different currencies, EUR and GBP. Are you aware that the transfermarkt data calculates past transactions at current exchange rate? How do you account for these exchange rate discrepancies?
- You might want to explain why you have chosen random forest and gradient boosting machine among the many machine learning models that are used as of today.
- Table 4 needs a makeover, it is hard to read and interpret. Having said that, the results of the MLR are not discussed. There is only a comparison between the models.

**Discussion**

- How do the results of the paper contrast to other studies that examined the impact of player popularity on transfer fees?
- Please detail the implications of this paper and your results for management?
