# Supplementary material for: Measuring the popularity of football players with Google Trends
Source: PLoS One. 2023 Aug 16;18(8):e0289213. doi: 10.1371/journal.pone.0289213 (PMC10431623; doi:10.1371/journal.pone.0289213)
Supplement: Attachment — Submitted filename: Response to Reviewers_10july.docx [file pone.0289213.s006.docx]

Prof. Dr. Florian Follert
Academic Editor
PLOS ONE

Valencia, 10th July 2023

Dear Prof. Follert,

We enclose the revised version of our article Measuring the popularity of football players with Google Trends *(ref. PONE-D-22-31349)* in which we have incorporated the referees’ changes and suggestions. We would like to thank the Reviewer for their insightful comments that have allowed us to really improve the original version of the paper.

Next, the changes made in the paper following the referee and editor recommendations are detailed and explained.

In addition, we also enclose written responses in which we give an answer to each of the points made in their reports.

Yours sincerely,

Pilar Malagón

**Response to Editor:**

We appreciate the changes and suggestions and your detailed reading of the paper. The changes made are detailed below.

1) **The more critical presentation of the so-called "market values" from the transfermarkt.de platform is essential. A broad literature uses these largely unreflectively as dependent variables in their models. I would like to ask you to take up the points of criticism.**

Following the recommendation of the editor, as well as the suggestion of referee 2, we have provided a more critical explanation of the Transfermarkt website. Page 5:

*“Numerous studies have acknowledged Transfermarkt as a useful approximation to players’ market value. However, such a crowdsourcing approach has its limitations. In this respect, M ̈uller et al. [7] point out that the objectivity of the community members, on whose opinions the valuation is based, cannot be guaranteed. The process of aggregating user valuations lacks homogeneity, making it difficult to reproduce the results. Moreover, the estimation of market value tends to have higher variability for less popular players receiving less attention and valuations. More recently, Franceschi et al. [5] stress the subjectivity already mentioned by [7] and emphasize the conceptual difference between the transfer fee and the estimated market value provided by platforms like Transfermarkt. Nevertheless, researchers have recognized the empirical proximity between these measures [5, 7, 13, 23, 26, 27].”*

**Response to referee 2:**

We appreciate the changes and suggestions indicated and your detailed reading of the paper. The changes made are detailed below.

1) **The final paragraph of the Introduction ("The remainder of the paper...") could be omitted as the manuscript adheres to a standard structure.**

We genuinely appreciate this comment, and the paragraph was omitted.

2) **It is worth noting that the use of market values has faced significant criticism from some academics. It would be advantageous to provide a more critical assessment and acknowledge the limitations associated with employing websites like www.transfermarkt.de. For instance, Müller et al. (2017) criticize the use of such platforms (https://www.sciencedirect.com/science/article/pii/S0377221717304332).**

Thank you for this precise comment. We have provided a more critical explanation of the Transfermarkt valuations. Page 5:

*“Numerous studies have acknowledged Transfermarkt as a useful approximation to players’ market value. However, such a crowdsourcing approach has its limitations. In this respect, M ̈uller et al. [7] point out that the objectivity of the community members, on whose opinions the valuation is based, cannot be guaranteed. The process of aggregating user valuations lacks homogeneity, making it difficult to reproduce the results. Moreover, the estimation of market value tends to have higher variability for less popular players receiving less attention and valuations. More recently, Franceschi et al. [5] stress the subjectivity already mentioned by [7] and emphasize the conceptual difference between the transfer fee and the estimated market value provided by platforms like Transfermarkt. Nevertheless, researchers have recognized the empirical proximity between these measures [5, 7, 13, 23, 26, 27].”*

3) **In their paper on the impact of Club revenues on transfer fees and salaries, Quansah et al. (2021) demonstrate that club revenues are a crucial variable in determining transfer fees and player market values, revealing the vulnerability of these factors during times of crises. Maybe it would be good to consider the implications of their findings for the relevance of utilizing Google Trends as a measure of popularity during periods of distress in your own research.**

Following this comment, we have included this concern as future research in the conclusions:

*“To provide a complete view of the impact of players' popularity on their market value, future research should also include longitudinal data to capture both economic growth and crisis periods, as club revenues could significantly influence market values [54].” – Page 28*

4) **Furthermore, it is mentioned that Cristiano Ronaldo is the most followed person on Instagram. Lionel Messi, another popular but « aging » football figure, closely trails him in terms of followers. While there certainly had been a strong correlation between popularity and market value earlier in their careers, it appears that this relationship has become nonlinear. Although their popularity continues to grow, their market values have experienced significant declines. Additionally, Messi's transfer from PSG to Inter Miami in the US, a relatively inferior league, has garnered substantial media attention and had a noteworthy impact on the player's popularity (and google trends), which may not be (or is certainly not) reflected in the player's continuously decreasing market value. While these are only two examples of anecdotal evidence, there are certainly more cases.**

This comment is really interesting. We agree that there is no linear relationship between popularity and market value. In fact, we believe that this is why the results of our work suggest that it is best studied with non-linear models.

5) **The paper includes several unsupported claims, such as "However, Google Trends has been underused and misused by sports analysts." It is essential to provide evidence or support for such claims rather than making assertions without a factual basis.**

Thank you for your comment. To address this concern, we have carefully revised the wording of the paper to focus on the underutilization of Google Trends in the context of sports analysis, rather than making assertions about misuse. Furthermore, we would like to emphasize that our proposed approach offers improved methods for utilizing Google Trends more effectively in the estimation of players' valuation.

Consequently, the referred sentence has been rewritten as: “However, Google Trends has been underused by sports analysts.”

**Similarly, the statement "The sports industry is a place where most interests, from political interests to public interests, converge, and traditional media and social media have helped to increase football's popularity worldwide" remains unsupported, along with certain other claims in the document. There are instances where the reader fails to discern the source of the information presented.**

Thank you for bringing this to our attention. We included the references used to support this affirmation in the revised version of the manuscript.

*“The sports industry is a place where most interests, from political interests to public interests, converge, and traditional media and social media have helped to increase football's popularity worldwide [1,2].” – Page 2*

[1] Galily, Y, Tamir, I, Limor, Y. Sports: Faster, Higher, Stronger, and Public

Relations. Hum. Aff. 2015 Mar;25(1):93–109.

[2] Seippel, Ø, Dalen, H B, Sandvik, M R, Solstad, G. M. From political sports to
sports politics: on political mobilization of sports issues. Int. J. Sport Policy
Politics. 2018 Mar;10(4):669–686.

6) **In the introduction, it is stated that the "Big Five" European football leagues generated revenues exceeding €9.283 million in the 2018/2019 season. As we are currently in 2023 and the 2022/23 season has concluded, it is necessary to update this information.**

Thank you for your comment; we have added to the text (page 2) the revenue generated in the 2022/2023 season. Also, please note that for the sake of consistency, we have kept the dates of the 2018/2019 season.

*“According to the consulting company Deloitte, the 20 clubs with the highest turnover (all of which belong to the ‘Big Five' European football leagues) exceeded €9.200 million in revenue in the 2021/22 season, marginally below pre-COVID revenues of €9.283 million in 2018/19 [3].” – Page 2*

7) **Additionally, the data used in the analysis pertains to the 2018/19 season. It is important to clarify the reason for analyzing such outdated data. Was it due to convenience, as the initial research was conducted four years ago?**

We appreciate the reviewer's comment on the appropriateness of the data. We would like to clarify that the database was taken in the summer of 2019 because these data were used for a master's thesis. Note that this work is framed in the context of different analyses that comprise a PhD thesis. Therefore, for reasons of research and consistency, all analyses (the study of leagues, matches, and players) have been carried out with data from the 2018-2019 season. In addition, we would like to express that, despite the undoubted interest in having up-to-date data, this work aims to develop and provide a new methodology for calculating several popularity indicators. In this way, although the study has been done using football players to the season 2018-2019, this paper provides practical guidance for developing and incorporating the proposed indicators, which could be applied in sports analytics and any study in which popularity is relevant. Therefore, we hope that this new methodology is tested by other researchers and by ourselves by means of a wide range of data.

8) **The statement "Thus, to solve the problem of the lack of information on the actual transfer fees, researchers and fans have started paying attention to websites that offer estimates of the market value of players" suggests that football fans have turned to websites like transfermarkt.de due to the scarcity of transfer fee information. It would be beneficial to provide evidence or support for this claim.**

Thank you again for pointing this out. Our references just support the use of these websites by researchers, so the mention to fans’ behavior has been removed. Citations to other research works using these websites have been included.

9) **The information regarding Cristiano Ronaldo's 308 million followers on Instagram may also be outdated? The last time I looked he had close to 600 million followers.**

Thanks for your suggestion. We have updated the information. Page 3

"*A clear example is Cristiano Ronaldo, who in 2021 had 308 million followers on Instagram (making him the person with the most followers in the world), charged $1.6 million per sponsored post, and earns more than 40 million dollars per year from publications alone [14]. Now, two years later, his followers exceed 590 million and he is still the person with the most followers in the world*."
